# P-765. Bactericidal activity of peracetic acid against common uropathogens: a potential bladder irrigant for self-catheterizing patients or those with indwelling catheters

**DOI:** 10.1093/ofid/ofaf695.976

**Published:** 2026-01-11

**Authors:** Daniel M Musher, Francisco X Elisarraras, Braydon Barrett, Donald P Griffith

**Affiliations:** Michael E. DeBakey VA Medical Center / Baylor College of Medicine, Houston, Texas; Baylor College of Medicine, Los Angeles, California; Baylor College of Medicine, Los Angeles, California; Baylor College of Medicine, Los Angeles, California

## Abstract

**Background:**

The presence of an indwelling urinary catheter and regular self-catheterization are highly associated with bacteriuria and the potential for clinically significant infection. Various irrigating solutions have been tried to prevent bacteriuria in such cases, with limited success. Peracetic acid (PA) is approved by the FDA for application to food products at 200 ug/mL. Use of this drug has not been reported for use in humans.

**Figure:**
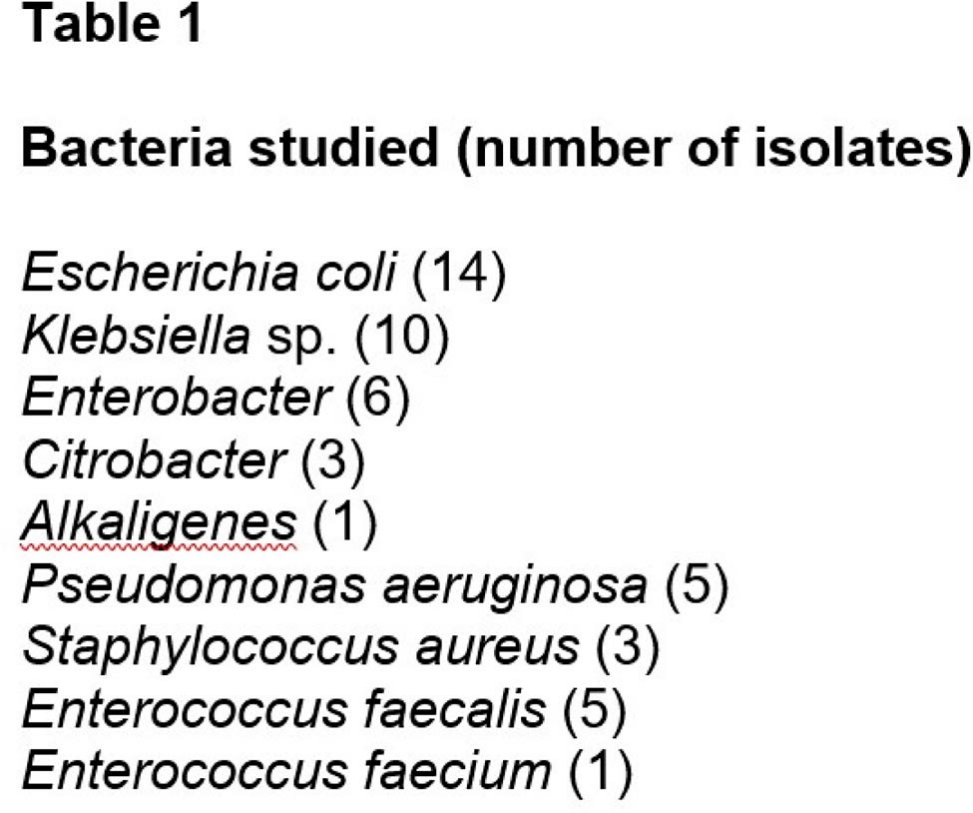

**Methods:**

Forty-four bacterial isolates from infected urine and 4 ATCC isolates were studied (see Table). Planktonic bacteria were studied after growth overnight in Mueller Hinton broth (MHB) at 37 C. Bacteria were added to serial dilutions of PA (3 to 150 ug/ml) to yield 2x10^6^ (Gram positive cocci) to 2x10^7^ (Gram negative rods) cfu/ml. Bacteria were quantitated after incubation at room temperature for 30 minutes. Biofilm was made by incubating bacteria in MHB in a gently swirling water bath at 37 C for 72 hr. Tubes were gently rinsed, and PA was added. Tubes were scraped with a ‘rubber policeman’ and bacteria were quantitated as above.

**Results:**

With 30 min exposure to PA, the MBC of all planktonic *Enterobacterales* was *<* 25 ug/ml, and the MBC_90_ of planktonic *Pseudomonas* and enterococci was < 25 ug/ml, with MBC of all strains < 50 ug/ml. Time-kill experiments showed that killing was extremely rapid. Because, in vivo, urine production would reduce concentration of PA, we studied killing by PA in a subset of 21 organisms after 5 min exposure to the same range of concentrations. MBC_90_ for *Enterobacterales* was < 25 ug/ml. Importantly, all organisms studied, whether planktonic or in biofilm state, were killed at 50 ug/ml.

**Conclusion:**

PA, at concentrations well below those that are sprayed on food products, was bactericidal for 48 organisms that cause UTI. This compound has not been used in human studies. In the absence of substances that safely and effectively suppress bacteriuria in patients who self-catheterize or have indwelling urinary catheters PA deserves consideration for future clinical trials.

**Disclosures:**

Donald P. Griffith, MD, None: Patent on peracetic acid + witch hazel is pending

